# How much heat does non-photochemical quenching produce?

**DOI:** 10.3389/fpls.2024.1367795

**Published:** 2024-03-20

**Authors:** Aoi Murakami, Eunchul Kim, Jun Minagawa, Kenji Takizawa

**Affiliations:** ^1^ Astrobiology Center, National Institutes of Natural Sciences, Osawa, Mitaka, Tokyo, Japan; ^2^ National Institute for Basic Biology, National Institutes of Natural Sciences, Nishigonaka, Myodaiji, Okazaki, Aichi, Japan; ^3^ Graduate Institute for Advanced Studies, SOKENDAI, Okazaki, Japan

**Keywords:** photosynthesis, non-photochemical quenching (NPQ), heat transfer, heat budget, global warming

## Abstract

Non-photochemical quenching (NPQ) is a protective mechanism used by plants to safely dissipate excess absorbed light energy as heat, minimizing photo-oxidative damage. Although the importance of NPQ as a safety valve for photosynthesis is well-known, the physiological and environmental effects of the heat produced remain unclear because the amount of heat produced by NPQ is considered negligible, and its physiological effects have not been directly observed. Here, we calculated the heat produced by NPQ and evaluated its impact on the leaf and global warming based on simplified models. Our evaluation showed that the heat produced by NPQ in a given leaf area is 63.9 W m^−2^ under direct sunlight. Under the standard condition, NPQ warms up the leaf at less than 0.1°C, but it could be 1°C under particular conditions with low thermal conductance. We also estimated the thermal radiation of vegetation’s NPQ to be 2.2 W m^−2^ par global averaged surface area. It is only 0.55% of the thermal radiation by the Earth’s surface, but still significant in the current climate change response. We further discuss the possible function of NPQ to plant physiology besides the safety valve and provide strategies with artificial modification of the NPQ mechanism to increase food production and mitigate global warming.

## Introduction

Green plants operate photosynthesis to convert light energy into chemical energy to produce organic compounds that support life on Earth. However, excessive light energy can lead to the production of reactive oxygen species and other harmful byproducts, which can damage photosynthetic machinery and impair photosynthetic efficiency (Niyogi, 1999). Non-photochemical quenching (NPQ) is a protective process through which plants safely dissipate excess absorbed light energy as heat, thereby minimizing photo-oxidative damage (Demmig-Adams et al., 2014). However, despite its importance as a safety valve for photosynthesis, the effects of the heat produced by NPQ on photosynthetic reactions and surrounding environments remain unclear. Although NPQ is a complex phenomenon involving a variety of processes and is defined differently depending on the measurement method and object of observation (Lazár, 2015), it is used here as a term for regulative heat dissipation mechanisms induced under light irradiation, including the quickly reversible component and long-sustained component (Verhoeven, 2014).

Green plants utilize atmospheric carbon dioxide to produce organic carbon. Evidently, carbon dioxide has a greenhouse effect (Hansen et al., 1981), so planting trees in the desert is expected to be a mitigation strategy to reduce global warming. However, afforestation has multiple biophysical effects, such as cooling by evaporation and a warming effect due to lower albedo, therefore, it can no longer be said to mitigate global warming in general (Bonan, 2008). The effect of these plants on cooling or warming against the climate varies depending on the climate, latitude, and many unknown aspects in microhabitat and physiological response.

In this article, we estimated heat produced by NPQ and explored its significance in terms of physiological and environmental impacts. We used a simplified model to calculate the heat produced by NPQ because of its complexity, which is influenced by various environmental and biological factors, including variations in solar radiation and physiological responses. Solar radiation reaching the Earth’s surface is affected by the altitude angle and weather, and the amount of light reaching the leaf varies in complex ways depending on the canopy structure and the leaf angle. Even under the same light intensity, NPQ varies depending on the other environmental conditions, plant type, growth stage, and physiological conditions. Here, we present the simple and reliable estimation of heat produced by NPQ and its possible and maximum effects on plant physiology and the environment.

By advancing our understanding of the role of heat by NPQ, we can contribute to the development of more efficient and sustainable agricultural practices and a broader understanding of the role of photosynthesis in the Earth’s ecosystems and environment.

## Energy conversion in photosynthesis and heat production by NPQ

**Figure 1:**
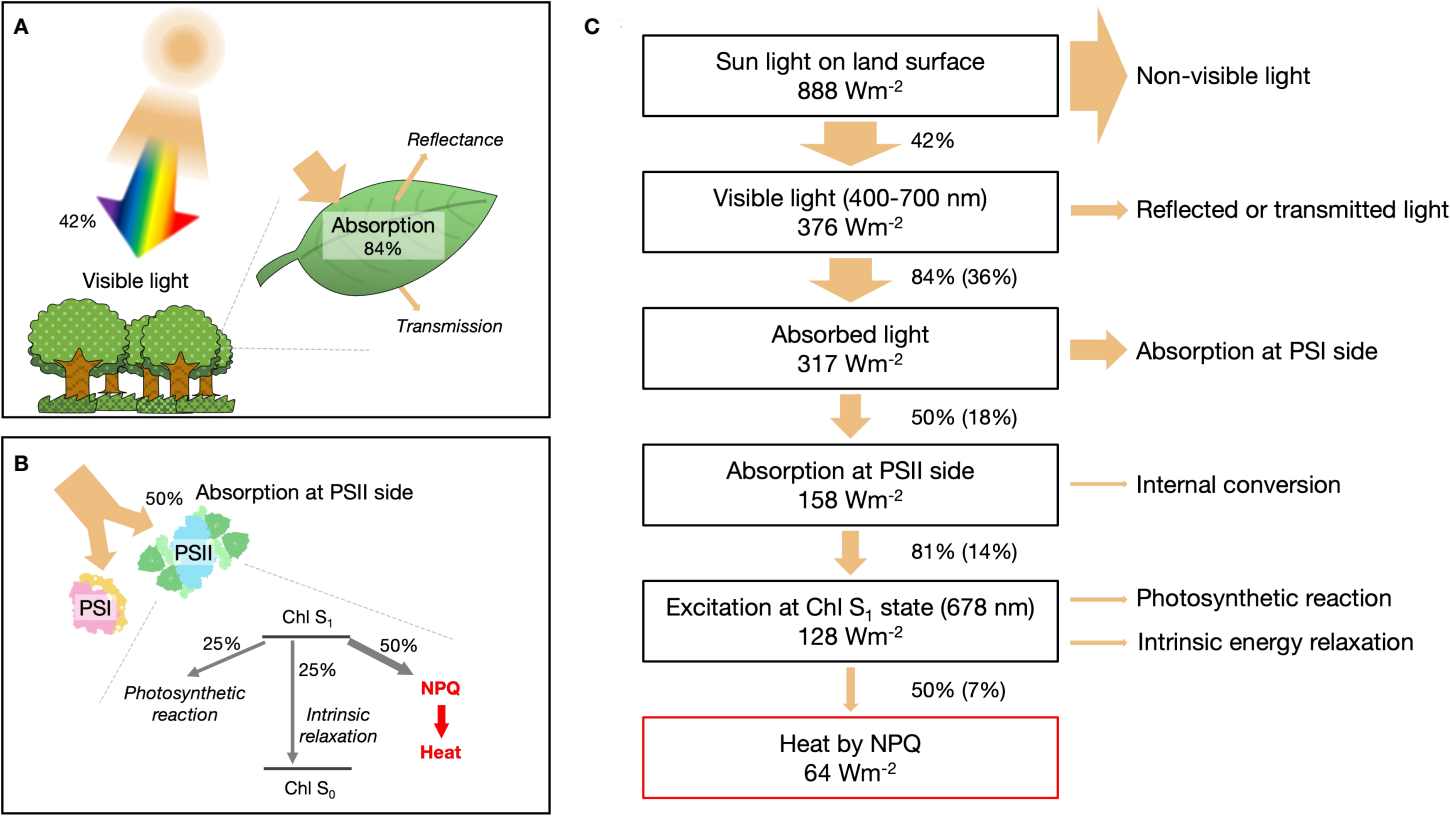
Quantitative assessment of heat generation *via* non-photochemical quenching (NPQ) on Earth. **(A)** Proportion of light energy absorbed by leaves. **(B)** Detailed quantification of light energy dissipation as thermal energy mediated by NPQ, emphasizing the involvement of photosystem I (PSI) and photosystem II (PSII) supercomplexes. **(C)** Schematic representation of the photonic energy conversion into thermal energy through NPQ processes on Earth.

Solar radiation reaching the Earth’s land surface, in terms of direct and circumsolar radiation, has a standard flux (ASTM G173-03) of 888 W m^−2^ (NREL, 2003). Visible light (400–700 nm) constitutes approximately 42.3% of this radiation and is the portion that can be absorbed by leaves (**Figure 1A**). In general, leaves can only capture approximately 84% of the available visible light (Björkman and Demmig, 1987). The absorbed energy is divided almost equally between the reaction centers in photosystem I (PSI) and photosystem II (PSII) (Björkman and Demmig, 1987) (**Figure 1B**). On the PSII side, the absorbed energy goes through three distinct pathways. First, charge separation occurs in the reaction center, facilitating the conversion of light energy into chemical energy. Second, intrinsic dissipation takes place within the chlorophyll. Finally, in the third pathway, excess energy is dissipated as heat through processes collectively referred to as NPQ, which regulates and protects the plant from potential damage (Li et al., 2009; Ruban et al., 2012).

Most of the absorbed light energy (317 Wm^-2^ in **Figure 1C**) besides NPQ is converted to heat. A small fraction of energy (≈35% at the maximum) of the charge separation in PSI and PSII is ultimately fixed as sugar; the rest is released as heat during the intermediate process. The intrinsic energy dissipation is also mostly thermal except for a few percent of fluorescence emission (Lazár, 2015). The amount of heat released by NPQ is less than that of the other processes combined, so it has not received much attention. We dare to focus on NPQ and examine in the subsequent sections whether its heat generation effect is negligible.

Under moderate direct sunlight, photosynthetic activity is saturated, and more than half of the excitation energy around PSII flows into the NPQ pathway (**Supplementary Figure S1**). In addition to light intensity, NPQ varies with factors such as temperature, CO_2_ concentration, and canopy structure. However, the general trend of previous experiments is for NPQ energy partitioning to be 40-60% when PSII quantum yield is 20-30% (Kramer et al., 2004; Melkonian et al., 2004; Guadagno et al., 2010; Chang et al., 2022). In this calculation, we assumed the yield of NPQ at PSII as 50% and the solar radiation corresponds to the midday in the average latitude for the contiguous USA. According to the simplified energy conversion model shown in **Figure 1C**, the amount of heat produced by NPQ is 63.9 W m^−2^, equivalent to 20.3% of absorbed light energy and 7.2% of solar radiation on the land surface.

## Increase in leaf temperature by NPQ

Plant leaves are known to keep their temperature appropriate for photosynthesis even in direct sunlight (Michaletz et al., 2016) through transpiration, convection, and radiation. The heating effect of NPQ has been considered negligible compared to the cooling effects. When the stomata is closed and there is no transpiration, leaf surface temperature significantly increase under illumination. The previous thermoimaging analysis of tobacco leaves showed the leaf temperature increase by 1-5°C, and the enhancing NPQ increased leaf temperature increase by 10% (Kana and Vass, 2008). Under these conditions, the inside leaf temperature could be higher than the surface temperature. However, the amount of heat accumulated in leaves has not been fully evaluated because of the lack of effective probes for sensing the temperature inside the leaf tissues and in a single cell.

**Figure 2:**
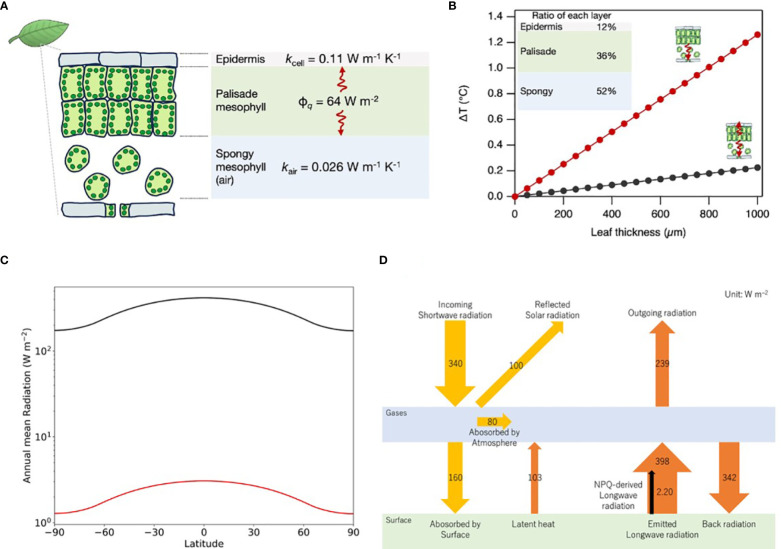
Thermal dynamics in leaf structures mediated by NPQ. **(A)** A one-dimensional geometrical model for the calculation of temperature variations induced by NPQ, incorporating the heat flux (ϕ*_q_*) and the thermal conductivities of the epidermal and spongy mesophyll (*k*_cell_ and *k*_air_, respectively). **(B)** Modeled temperature variations as a function of the leaf thickness, incorporating empirical layer proportion data derived from the *Arabidopsis* study (Gorsuch et al., 2010). Black and red lines represent two situations respectively, (i) the heat transfers to both abaxial (spongy layer) and adaxial (epidermis layer) sides; (ii) the heat transfers only to the abaxial side when the adaxial side is hotter than the abaxial side due to direct sunlight irradiation. **(C)** Variation of annual mean radiation at different latitudes. The black line is SWR and the red line is NPQ-induced LWR. **(D)** Schematic of Earth’s global average energy budget incorporating NPQ-induced LWR with reference to IPCC’s AR6.


We assumed leaf condition to form a maximum temperature difference between the outside, closed stomata, no transpiration, and no convection in the intercellular spaces. In this case, the heat transfer is evaluated on the 1D-geometry model, as shown in **Figure 2A**, using the simplified Fourier’s heat transfer equation:

$$\Delta T=\phi_{q}\frac{dx}{k}$$

Where *ΔT*, ϕ*_q_*, *k*, and *dx* are the temperature difference (K), heat flux (W m^−2^), thermal conductivity (W m^−1^ K^–1^), and length of heat transfer (m) respectively.

A typical leaf tissue structure consists of a tightly packed palisade mesophyll layer on the upper (adaxial) side and a spongy mesophyll layer on the lower (abaxial) side (**Figure 2A**). When we assume that the heat produced by NPQ is mainly generated in the palisade layer, the value of ΔT formed by abaxial side heat transfer *via* cells and adaxial side heat transfer *via* air (ΔT_abaxial_+ΔT_adaxial_=ΔT_leaf_) can be driven from the following equation:


$$\Delta T_{leaf}=\phi_{q}/\left(\frac{k_{cell}}{d_{cell}}+\frac{k_{air}}{d_{air}}\right)$$

Assuming the relative thickness of the upper epidermis, palisade, and spongy mesophyll to be 12%, 36%, and 52%, respectively, as in the case of *Arabidopsis* (Gorsuch et al., 2010), ΔT_leaf_ increases with increasing leaf thickness, as shown in the graphs of **Figure 2B**. When the heat in palisade cells transfers to the abaxial and adaxial sides, the ΔT_leaf_ is 0.06°C (leaf thickness ~ 250 µm in the case of *Arabidopsis* leaf), even when using a relatively low value of cellular thermal conductivity at 0.11 W m^–1^ K^–1^ (Sotoma et al., 2021). On the other hand, when the heat is transferred only to the abaxial side, ΔT_leaf_ increases by 0.3°C because of the very low thermal conductivity of the air at 0.026 W m^–1^ K^–1^. Such a situation is conceivable when the ambient temperature of the abaxial side is lower than the temperature of the adaxial side, and the leaf temperature is in between either of them. In the case of thick leaves at 1 mm (e.g. evergreen trees), leaf tissue can hold a temperature gradient of about 1°C.

Although the above calculations show that NPQ could increase leaf tissue temperature by 1°C at the maximum under the extreme conditions, the temperature inside the chloroplast could be much higher. The temperature dynamics and subcellular variation inside living cells have been obscure for a long time. In mammalian cells, recently developed nanothermometry probes enable the measurement of intracellular temperature. It is revealed that the mitochondrial temperature could increase by 10°C by stimulating respiration, which is 10^5^ times higher than the theoretical estimation (Chrétien et al., 2018; Di et al., 2022). Whereas typical animal cells with a radius of 10 µm generate 1 nW of heat per cell (Hong et al., 2020) by consuming sugars and lipids, a plant cell of the same cross-section area can continue to generate 10 times as much heat by NPQ under continuous solar illumination (314.2 µm^2^ × 63.9 W m^–2^ = 20.1 nW). Since photosynthetic electron transport occurs in the thylakoid membranes placed in chloroplast stroma, which is surrounded by a double membrane envelope, they might retain a large amount of heat locally if the membrane structures can prevent heat exchange. Indirect observation of heat emission from photosynthetic systems via photoacoustic techniques has ruled out the existence of heat barriers inside and surrounding the chloroplast (Mullineaux et al., 1994). The development of technology to directly and accurately measure inner cell temperature distribution, along with theoretical calculations considering the three-dimensional complexity of organelles and cellular arrangements within leaves is awaited.

## Increase in global temperature by NPQ

The Earth’s thermal equilibrium is determined by the balance between the incoming shortwave (300-3,000 nm) radiation (SWR) from the sun and the outgoing longwave (5-25 μm) radiations (LWR) from the land surface and the atmosphere. The development of land vegetation not only has a cooling effect as it absorbs atmospheric CO_2_ and latent heat by transpiration but also has a warming impact due to the absorption of light energy and its conversion to heat. In this section, we calculated the share of NPQ in the global heat budget and estimated the effect on global temperature.

In previous sections, we calculated that the heat produced by NPQ is 63.9 W m^–2^ at noon in the mid-latitude, where the solar zenith angle is 48.19 degrees. This section evaluates thermal radiation by NPQ globally by considering changing solar zenith angle by diurnal cycle, annual cycle, and latitude. Time-averaged SWR at each latitude of the Earth (Q(ϕ)) is calculated using Python’s climlab package (Rose, 2018), as explained in the **Supplementary Material** and shown in **Figure 2C**. Given the Q(ϕ), NPQ-derived fraction of LWR (I_NPQ_(ϕ)) can be obtained from the following equation:


$$I_{NPQ}\left(\phi \right)=\text{Q}\left(\phi \right)\cdot t_{a}\cdot r_{land}\cdot r_{veg}\cdot r_{NPQ}\cdot \varepsilon_{veg}$$

Where t_a_, r_land_, r_veg_, r_NPQ_, and ε_veg_ are atmospheric transmittance, the ratio of land area to global area, the ratio of vegetation area to the land area, the ratio of NPQ thermal energy to solar radiation on the land surface, and emissivity of vegetation respectively. t_a_ is estimated to be 0.47 from the ratio of the surface irradiance at 160 W m^-2^ to the incoming solar irradiance at 340 W m^-2^ (IPCC, 2021). r_land_ is 0.30, as generally known. r_veg_ is estimated at 0.74 from **Supplementary Table S1** (FAO, 2023). r_NPQ_ was calculated to be 0.07 in the previous section. Emissivity values change depending on the material. Typically, the emissivity for water is between 0.98 and 0.99 (Konda et al., 1994), soil is between 0.96 and 0.97, and vegetation is between 0.96 and 0.98 (Qin et al., 2006). For simplicity, we used the same values at 0.98 for vegetation and global average. The result of this calculation is shown in **Figure 2C**.

A weighted average of I_NPQ_ (ϕ) for each latitude (ϕ *_i_*) from 90 degrees north to 90 degrees south gives a global average value (I_NPQ_^global^).


$${I_{\mathrm{NPQ}}}^{\mathrm{global}}=\frac{\sum_{i=1}^{180}\left(I_{NPQ}(\phi_{i}\right)\cdot cos\phi_{i})}{\sum_{i=1}^{180}\left(cos\phi_{i}\right)}$$

The calculation results in 2.20 W m^–2^ for I_NPQ_^global^. The global average of Q (ϕ) calculated from the weighted average is at 341 W m^-2^, consistent with the latest estimation by IPCC at 340 W m^-2^ (IPCC, 2021). Our estimation of NPQ-induced LWR at 2.20 W m^–2^ occupies 0.55% of the LWR from the land surface at 398 W m^-2^ estimated by IPCC (see **Figure 2D**).

Finally, the relationship between the thermal radiation by NPQ and the Earth’s surface temperature is calculated from Stefan-Boltzmann’s law in the following equation:


$$I=\varepsilon \sigma T^{4}\;\Leftrightarrow \;T={\left(\frac{I}{\varepsilon \sigma }\right)}^{\frac{1}{4}}$$

Where I, ε, σ, and T are radiant emittance (W m^–2^), emissivity (0.98), Stefan–Boltzmann constant (5.67×10^–8^ W m^–2^ K^–4^), and temperature (K), respectively. Considering that LWR from the land surface at 398 W m^–2^, the surface temperature is 290.9 K (17.8°C). If the radiant emittance corresponding to NPQ (2.2 W m^-2^) is removed, the surface temperature decreases by 0.4 K to 290.5 K. The result indicates that NPQ contributes to an increase in the Earth’s surface temperature of 0.4°C.

## Discussion

Heat dissipation by NPQ is a primary feedback regulation mechanism in photosynthesis under excessive light, and its mechanism has been intensely studied for decades (Bassi and Dall’Osto, 2021). NPQ is also a direct target for crop engineering to increase food and energy production since it is considered wasted energy. However, simply suppressing NPQ will only substitute excess energy for constitutive non-photochemical quenching (light-independent) heat dissipation, not carbon fixation (see **Supplementary Figure S1**). Mutational analyses revealed that NPQ-less plants and algae could grow well under low light but were less adaptive to high light and changing light conditions (Khuong et al., 2019; Perin et al., 2023). A faster off-switching of unnecessary NPQ can significantly improve crop production only under fluctuating light (Kromdijk et al., 2016; De Souza et al., 2022). Constant suppression of NPQ and improvement of photosynthetic productivity has not been successful so far. To increase the photosynthetic productivity of plants under natural conditions, NPQ suppression must be accompanied by improved energy utilization. It would require extensive genetic modification to regulate multiple targets downstream of the light reactions including dark reactions, gas exchange, carbon translocation, and sink capacity.

Conversely, we cannot ignore the possibility that thermal energy, including NPQ, is involved in the mechanisms to optimize photosynthetic functions. Illumination of dark-adapted plants induces a pronounced thermal energy dissipation and activates the light-harvesting antenna complex, transferring excitations toward the reaction centers (Janik et al., 2017). Moreover, the direct use of thermal energy via the up-conversion of excitation energy has been demonstrated experimentally (Zubik et al., 2020).

If NPQ significantly raises the intracellular temperature, it could affect biochemical and physiological reactions in photosynthesis. Heat production should be beneficial for plants to protect them from chilling stress. Snow algae and some cold-tolerant plants can perform photosynthesis under sub-freezing temperatures. It could be possible that these plants maintain local temperatures around photosynthetic reaction centers. Evergreen plants from high latitudes can sustain high rates of NPQ during winter (Verhoeven, 2014). The Antarctic psychrophile green algae temporally induce a high NPQ during the induction of photosynthesis (Takizawa et al., 2009). Heat production by NPQ might warm up the photosynthetic machinery to enable quick induction of photosynthesis at low temperatures.

Thermal production at moderate and high temperatures does not accelerate photosynthesis but might downregulate it safely. High temperature and high light stress often co-occur, and they can induce similar photoprotective mechanisms, such as state transitions (Nellaepalli et al., 2011) and activation of cyclic electron flow (Sharkey, 2005). NPQ might enhance high temperature-induced photoprotection under moderate ambient temperature. Once it can be confirmed that heat production by NPQ does not affect photosynthetic activity and plant physiology, a large-scale gene editing project to suppress NPQ can be initiated.

The artificial control of NPQ can also be used as a strategy to modulate global temperature. The radiation from plant NPQ is about 2.2 W m^–2^ par surface area of the earth. It is less than 1% of the thermal radiated from the Earth’s surface, but it cannot be ignored. The climate forcing caused by the doubling of atmospheric CO_2_ by human activities has perturbed Earth’s energy balance about 4 W m^–2^ (Wilson and Gea-Banacloche, 2012; Jeevanjee et al., 2021). Theoretically, about half of this could be offset by suppressing NPQ. To cool the Earth, suppression of NPQ must be coupled with suppression of the light capture because once light is absorbed by the plant, almost all of it is finally converted to heat. Increasing leaf reflectance reduces the light available for photosynthesis, but plants with suppressed NPQ may be able to grow adequately.

The environment and living organisms have coevolved in the Earth’s long history. The evolution and expansion of terrestrial plants could have made the environment cooler and caused the Ice Age (Richey et al., 2020). However, plants with NPQ could also contribute to global warming or mitigating global cooling. Most existing plants adapt to their environment by absorbing light efficiently and letting excess energy escape as heat. If plants that reflect excess light and use less light come to occupy the planet, the global environment may become colder.

## Data availability statement

The original contributions presented in the study are included in the article/**Supplementary Material**. Further inquiries can be directed to the corresponding author/s.

## Author contributions

AM: Investigation, Writing – original draft, Writing – review & editing. EK: Investigation, Writing – original draft, Writing – review & editing. JM: Conceptualization, Writing – review & editing. KT: Investigation, Project administration, Writing – original draft, Writing – review & editing.
