# Supplementary material for: How much heat does non-photochemical quenching produce?
Source: Front Plant Sci. 2024 Mar 20;15:1367795. doi: 10.3389/fpls.2024.1367795 (PMC11027892; doi:10.3389/fpls.2024.1367795)
Supplement: Supplementary file 1 [file DataSheet_1.pdf]

frontiers

Supplementary Material

1 Supplementary Equations

Time-averaged SWR at each latitude of the Earth (Q(ϕ))

By incorporating the expression for the solar zenith angle into the equation for insolation, we obtain a formula that allows us to calculate the instantaneous solar radiation received at any given latitude, during any season, and at any moment of the day:

$$Q(\phi) = S_0\left(\frac{\bar{d}}{d}\right)^2 \cos\theta_s \ = S_0\left(\frac{\bar{d}}{d}\right)^2 (\sin\phi\sin\delta + \cos\phi\cos\delta\cos h)$$

Where Q, S0, d, θs, φ, δ, and h are instantaneous radiation (W m^{-2}), solar constant (1366 W m^{-2}), distance between Sun and Earth, Solar zenith angle, latitude of observation point (-90 to 90 degrees), Solar declination angle and Solar hour angle. In this calculation, φ was sliced off every 180 degrees, and calculations were performed for every 1 degree.

The daily mean is obtained from the above equation by integrating the hourly angle from sunrise to sunset and dividing by 24 hours (i.e., 2π radians, which is one revolution):

$$\overline{Q_i}^{day}(\phi) = \frac{1}{2\pi}\int_{-h_0}^{h_0} Q(\phi)\, dh = \frac{S_0}{2\pi}\left(\frac{\bar{d}}{d}\right)^2 \int_{-h_0}^{h_0} (\sin\phi\sin\delta + \cos\phi\cos\delta\cos h)\, dh$$

Q(ϕ) represents the annual mean for each latitude and was obtained from the daily mean by the following equation:

$$Q(\phi) = \frac{1}{365}\sum_{i=1}^{365} \overline{Q_i}^{day}(\phi)$$

2 Supplementary Tables

Table S1. The fraction of vegetated areas and other areas identified by ESA's CCI-LC in 2020 (FAO, 2023). The ratio of vegetation area to the land area (r_{veg}) was calculated as the ratio of Vegetated cover to Total cover.

	Land cover	Area (km^2)
Vegetated cover	Herbaceous crops	19,041,364
	Woody crops	2,224,764
	Grassland	18,150,069
	Tree-covered areas	42,682,690
	Mangroves	184,262
	Shrub-covered areas	16,056,588
	Shrubs and/or herbaceous vegetation, aquatic or regularly flooded	1,931,696
	Sparsely natural vegetated areas	8,890,803
	Multiple or layered crops	-
No-vegetated cover	Artificial surfaces (including urban and associated areas)	604,972
	Terrestrial barren land	19,129,863
	Permanent snow and glaciers	14,375,745
	Inland water bodies	3,829,139
	Coastal water bodies and intertidal areas	-
	Total cover	147,101,956

Food and Agriculture Organization of the United Nations (FAO). (2023). ESA's climate change initiative land cover (CCI-LC). https://www.fao.org/faostat/en/#data/LC [Accessed July 21, 2023]

3. Supplementary Figure

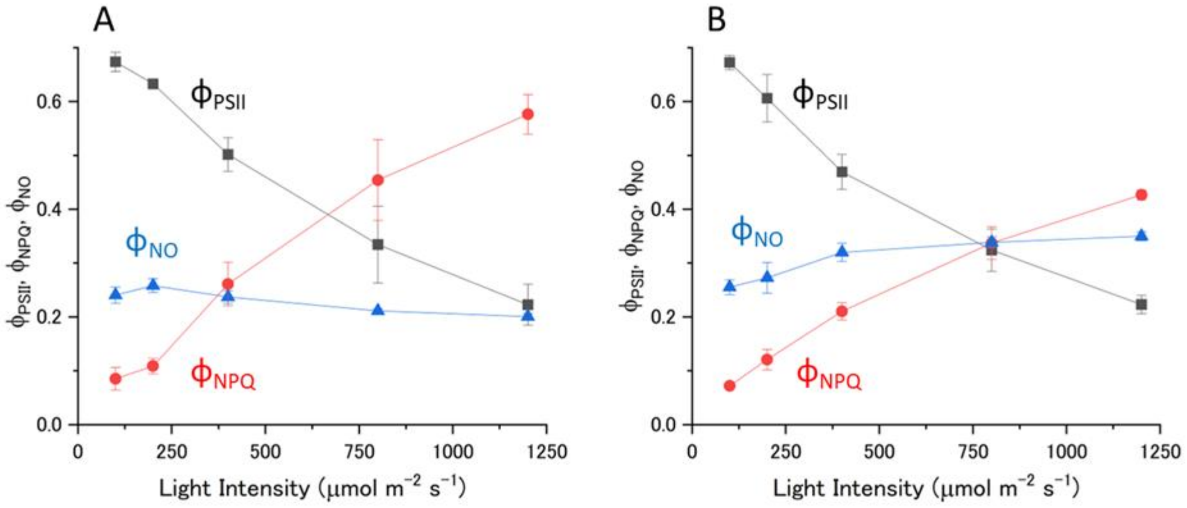

Figure S1. Light intensity-dependent changes in quantum yield of PSII for photochemistry (ϕ_{PSII}), NPQ (ϕ_{NPQ}), and other energy losses (ϕ_{NO}). Chlorophyll fluorescence from *Arabidopsis thaliana* leaves was measured by PAM 101 spectrometer under xenon lamp illumination at various intensities, and parameters were calculated as described by Kramer et al. (2004) (n=3, ±sd). **(A)** In the wild type, increasing light intensity decreased ϕ_{PSII} and increased ϕ_{NPQ}. ϕ_{NO} was not much changed. It suggests that the excessive light energy mostly flowed into NPQ. **(B)** In the NPQ-suppressed mutant, npq4 (Li et al., 2000), increasing light intensity decreased ϕ_{PSII} as in WT, but ϕ_{NPQ} was not as much increased as in the wild type. Increasing light significantly increased ϕ_{NO}, suggesting that the excessive light energy flowed into fluorescence and heat dissipation mechanisms other than NPQ.

Kramer, D.M., Johnson, G., Kiirats, O., and Edwards, G.E. (2004). New fluorescence parameters for the determination of Q_A redox state and excitation energy fluxes. *Photosynthesis Research* 79(2)**,** 209-218. doi: DOI 10.1023/B:PRES.0000015391.99477.0d.

Li, X.P., Bjorkman, O., Shih, C., Grossman, A.R., Rosenquist, M., Jansson, S., et al. (2000). A pigment-binding protein essential for regulation of photosynthetic light harvesting. *Nature* 403(6768), 391-395. doi: Doi 10.1038/35000131.
